# Can a Single Variable Predict Early Dropout From Digital Health Interventions? Comparison of Predictive Models From Two Large Randomized Trials

**DOI:** 10.2196/43629

**Published:** 2023-01-20

**Authors:** Jonathan Bricker, Zhen Miao, Kristin Mull, Margarita Santiago-Torres, David M Vock

**Affiliations:** 1 Division of Public Health Sciences Fred Hutch Cancer Center Seattle, WA United States; 2 Department of Psychology University of Washington Seattle, WA United States; 3 Department of Statistics University of Washington Seattle, WA United States; 4 Division of Biostatistics University of Minnesota Minneapolis, MN United States

**Keywords:** acceptance and commitment therapy, ACT, attrition, digital interventions, dropout, eHealth, engagement, iCanQuit, mobile health, mHealth, QuitGuide, smartphone apps, smoking, tobacco, trajectories, mobile phone

## Abstract

**Background:**

A single generalizable metric that accurately predicts early dropout from digital health interventions has the potential to readily inform intervention targets and treatment augmentations that could boost retention and intervention outcomes. We recently identified a type of early dropout from digital health interventions for smoking cessation, specifically, users who logged in during the first week of the intervention and had little to no activity thereafter. These users also had a substantially lower smoking cessation rate with our iCanQuit smoking cessation app compared with users who used the app for longer periods.

**Objective:**

This study aimed to explore whether log-in count data, using standard statistical methods, can precisely predict whether an individual will become an iCanQuit early dropout while validating the approach using other statistical methods and randomized trial data from 3 other digital interventions for smoking cessation (combined randomized N=4529).

**Methods:**

Standard logistic regression models were used to predict early dropouts for individuals receiving the iCanQuit smoking cessation intervention app, the National Cancer Institute QuitGuide smoking cessation intervention app, the WebQuit.org smoking cessation intervention website, and the Smokefree.gov smoking cessation intervention website. The main predictors were the number of times a participant logged in per day during the first 7 days following randomization. The area under the curve (AUC) assessed the performance of the logistic regression models, which were compared with decision trees, support vector machine, and neural network models. We also examined whether 13 baseline variables that included a variety of demographics (eg, race and ethnicity, gender, and age) and smoking characteristics (eg, use of e-cigarettes and confidence in being smoke free) might improve this prediction.

**Results:**

The AUC for each logistic regression model using only the first 7 days of log-in count variables was 0.94 (95% CI 0.90-0.97) for iCanQuit, 0.88 (95% CI 0.83-0.93) for QuitGuide, 0.85 (95% CI 0.80-0.88) for WebQuit.org, and 0.60 (95% CI 0.54-0.66) for Smokefree.gov. Replacing logistic regression models with more complex decision trees, support vector machines, or neural network models did not significantly increase the AUC, nor did including additional baseline variables as predictors. The sensitivity and specificity were generally good, and they were excellent for iCanQuit (ie, 0.91 and 0.85, respectively, at the 0.5 classification threshold).

**Conclusions:**

Logistic regression models using only the first 7 days of log-in count data were generally good at predicting early dropouts. These models performed well when using simple, automated, and readily available log-in count data, whereas including self-reported baseline variables did not improve the prediction. The results will inform the early identification of people at risk of early dropout from digital health interventions with the goal of intervening further by providing them with augmented treatments to increase their retention and, ultimately, their intervention outcomes.

## Introduction

### Background

Digital interventions, including smartphone apps, websites, and SMS text messaging interventions, have proven efficacious for a wide variety of behavioral health outcomes, including mental health, diet, exercise, and smoking cessation [1-6]. An important value of digital interventions for behavioral health is that they are accessible at a low cost, thereby increasing their potential public impact and reducing disparities in behavioral health care [7-9]. An ongoing central challenge to the overall efficacy and broadly scalable implementation of digital interventions is the phenomenon of users dropping out early [10]. Although *early drop out* has been variously defined across studies (eg, completing only 1 module or stopping within the first week or the first few weeks) [10,11], the fraction of users who fall into this category is quite large, with some studies showing it as high as 82% [12-14]. The fact that early dropouts are strongly associated with poor treatment success and constitute a large proportion of users substantially limits the potential public health impact of digital interventions.

A growing body of empirical literature aims to address this problem by examining variables that might predict early dropout and then comparing statistical models that improve the precision of these predictions [12-20]. The premise of this line of research is that, if users who are at a high risk of dropping out early can be identified, researchers and intervention developers can potentially augment the intervention (eg, proactive outreach, personalized human coaching, and tailored messaging) with the goal of improving retention and overall treatment efficacy. Providing augmented interventions only to potential early dropouts saves valuable resources for those who need them the most. The types of variables that have been used to predict early dropout have included self-reported baseline data (eg, demographics); objective measures of intervention engagement (eg, number of log-ins and proportion of content completed); and even sophisticated variables of user journeys, which encompass the myriad of sequences of interactions that a user takes to navigate through a digital intervention program [21]. Statistical methods for predicting early dropout have ranged from standard logistic and proportional hazard regression models to decision trees, random forests, and other machine learning techniques [22]. The results of these studies have widely varying discriminative ability, and the predictor variables were often idiosyncratic to the intervention (eg, number of intervention forum visits or participation badges obtained). In addition, multiple predictors were required for good discrimination, and some of the analyses required complex statistical models. For example, a study testing 7 machine learning models using 36 baseline variables found poor predictive performance of early dropout from an eating disorder digital intervention tested in 3 randomized trials (N=826), although adding multiple measures of intervention use from a subsample improved model prediction [15]. Another study of 2684 patients that predicted dropout from an eHealth lifestyle intervention found that a random forest model of 11 predictor variables was more accurate than similar models using logistic regression and decision trees, although many of the predictor variables were unique to the intervention (eg, intervention provider and number of times the interventionist provided advice) [20]. Other studies have similarly used a varying blend of baseline factors and use variables to calculate the probability of intervention attrition [23]. Taken together, a major gap in this literature is the identification of a small number of highly generalizable and easily collected variables that can predict early dropout with high precision tested in statistical models that can be readily implemented by other researchers and intervention developers. This study aimed to address this gap.

Recently, we published 2 studies identifying a type of early dropout from digital interventions that is highly predictive of treatment outcomes [24,25]. Specifically, we examined functional clustering longitudinal patterns of intervention use in a randomized trial (N=2415) that compared 2 conceptually distinct smartphone apps for smoking cessation (ie, iCanQuit and the National Cancer Institute [NCI]’s QuitGuide) and in a trial (N=2637) that compared 2 conceptually distinct websites for smoking cessation (WebQuit.org and Smokefree.gov). In both trials, the main treatment outcome was smoking cessation (ie, 30-day point prevalence abstinence) at the 12-month follow-up. Functional clustering analyses of these 4 interventions consistently identified a type of early dropout. The advantage of the functional clustering approach is that it is data rich, meaning that it accounts for the entire period of longitudinal follow-up—as opposed to use on any given day. Thus, this analytic approach can characterize a cluster of users by their unique longitudinal pattern of use, thereby allowing us to identify an important cluster of users, approximately half of all participants across all 4 interventions, who logged in during the first week and had little to no activity thereafter. Smoking cessation rates were consistently the lowest among the 1-week users compared with the other trajectory groups that had substantial log-in activity over time. For example, our iCanQuit smartphone app for smoking cessation based on acceptance and commitment therapy (ACT) had a 23% cessation rate among 1-week users compared with a 30% cessation rate for 4-week users (odds ratio 1.50, 95% CI 1.05-2.14; *P*=.03). Such differences in cessation rates are substantial, especially when considering that, across all 4 interventions, 49%-65% of all users were 1-week users and indeed were the largest log-in trajectory group [24,25]. The ability to prospectively predict who will become a 1-week user could be highly valuable as it would provide the opportunity to intervene early with an augmented treatment.

### Objectives

The goal of this study was to explore whether log-in count data using standard statistical methods can predict whether an individual will become an iCanQuit early dropout (ie, 1-week user) while validating the approach using the randomized trial data from the 3 other digital interventions for smoking cessation (combined N=4529), namely, QuitGuide, WebQuit.org, and Smokefree.gov. We chose log-in count data from each of the first 7 days following random assignment to the treatment arm as log-ins are a simple metric that is objectively collected without requiring user self-report and is readily available to both researchers and developers deploying interventions in real-world contexts (eg, on the Google Play and Apple App Stores). We also examined whether 13 baseline variables that included a variety of demographics (eg, race and ethnicity, gender, and age) and smoking characteristics (eg, use of e-cigarettes and confidence in being smoke free) might improve this prediction. The potential value of this study is to focus limited resources on the development and testing of augmented intervention components that could be offered only to those at high risk of early dropout, thereby improving both their use of the intervention and, in turn, their treatment outcomes.

## Methods

### Design

For the main aim of training a model to predict early dropouts, data were drawn from the iCanQuit app arm of a 2-arm randomized controlled trial (RCT) comparing iCanQuit with the NCI QuitGuide app for smoking cessation, with full protocol details previously described [26]. In brief, the trial included a racially and ethnically diverse sample of 2415 adult daily smokers from all 50 US states who were randomized 1:1 to receive access to an ACT-based smartphone app (iCanQuit; n=1214) or a United States Clinical Practice Guidelines (USCPG) approach–based smartphone app (QuitGuide; n=1201) for smoking cessation [27].

For validating the statistical approach predicting early dropouts across interventions, data were drawn from the QuitGuide app arm of the aforementioned RCT [26] as well as from the WebQuit.org and Smokefree.gov websites tested in a separate randomized trial [28]. In brief, this second trial also included a racially and ethnically diverse sample of 2637 adult daily smokers from all 50 US states who were randomized 1:1 to receive access to an ACT-based smoking cessation website (WebQuit.org; n=1319) or a USCPG-based website (Smokefree.gov; n=1318) for smoking cessation.

The eligibility criteria for each trial, fully described in each trial’s main outcome paper [26,28], were very similar overall: participants had to be adult smokers in the United States (aged ≥18 years) who smoked at least 5 cigarettes daily, and were motivated to quit smoking in the next 30 days. Recruitment, enrollment, and follow-up methods were identical *across the 2 RCTs* and, thus, were fully comparable across each of the 4 interventions [26,28]—participants were recruited nationally via Facebook advertisements, a survey sampling company, search engine results, or friend or family referrals. Participants completed an encrypted web-based screening survey and were notified of their eligibility via email. They then clicked on their secured emailed link to the study website, where they provided consent and completed the baseline survey. At each enrollment step, each study was presented as a comparison of 2 digital programs for smoking cessation. Participants could access their randomly assigned interventions from the moment of randomization and beyond (ie, after the 12-month follow-up period).

### Ethics Approval

All study activities for both trials were approved by the Fred Hutchinson Cancer Center Institutional Review Board (approval numbers: IR-8317/RG1001191 for iCanQuit; IR-7859/RG100176 for WebQuit). Participants provided consent on the web by clicking an “I accept” button option on the web-based consent form. All data were deidentified and collected on a secure web-based database. Participants received US $25 for completing each follow-up survey and an additional US $10 bonus if the web-based survey was completed within 24 hours of the initial email invitation to take the survey.

### Digital Interventions

#### Overview

Full descriptions of each digital intervention, which were double-blinded, are provided in their respective RCT main outcome papers [26,28] along with engagement summary statistics. The mean total number of log-ins to iCanQuit, QuitGuide, WebQuit, and Smokefree was 37.5 (SD 88.4), 9.9 (SD 50.0), 9.2 (SD 29.9), and 5.1 (SD 11.9), respectively, at the 12-month follow-up. The recommended use of all 4 interventions was daily for 6 weeks, allowing for 2 weeks of prequitting planning and 4 weeks of postquitting support for relapse prevention. Beyond that, all participants could keep returning to their assigned interventions as needed for ongoing support—as they would typically do in a real-world self-help intervention. In addition, as in any real-world self-help intervention, participants could go faster or slower or use their intervention for a longer or shorter period than recommended. No medications to aid cessation or other support were provided for any of the interventions, although all interventions provided education on Food and Drug Administration–approved medications for smoking cessation.

The overall differences between the 4 interventions were that (1) iCanQuit and WebQuit taught skills based on the principles of ACT [29] for smoking cessation [30-34], whereas QuitGuide and Smokefree taught skills based on the USCPG for smoking cessation [27], and (2) iCanQuit and QuitGuide were smartphone apps, whereas WebQuit and Smokefree were websites. Each intervention is briefly described in the following sections.

#### iCanQuit

The iCanQuit smartphone app intervenes in the ACT-focused processes of acceptance of internal cues to smoke and enacting one’s values that guide smoking cessation [26]. The acceptance component of the app teaches skills to accept physical sensations, emotions, and thoughts that trigger smoking via distancing oneself from thoughts about smoking (“cognitive defusion”), mindfulness skills, and flexible perspective taking. The values component of the app teaches skills for determining the core life domains that motivate smoking cessation (eg, family, health, and spirituality) and taking repeated small actions within these domains (eg, playing with grandchildren) to develop a smoke-free life. The program is self-paced, and the content is unlocked in a sequential manner across 8 levels. Each of the first 4 levels is accessible immediately after the previous level is completed, whereas each of the last 4 levels is only unlocked upon recording 7 consecutive days without smoking.

#### QuitGuide

The US NCI QuitGuide smartphone app content is delivered in four main sections: (1) “Thinking about quitting,” which focuses on motivations to quit by using reason and logic, such as identifying reasons to quit and providing information on the health consequences of smoking and quitting; (2) “Preparing to Quit,” which helps users develop a quit plan, identify smoking behaviors and triggers as well as reasons for being smoke free, and social support for quitting; (3) “Quitting,” which teaches skills for avoiding cravings to smoke; and (4) “Staying Quit,” which presents tips, motivations, actions to stay smoke free, and skills for coping with slips.

#### WebQuit

The WebQuit website is based on ACT [28], an approach that teaches skills to smokers to let their urges pass without smoking. The program has 4 parts that are funneled in sequence (ie, they follow a structured path). Step 1, “Make a Plan,” allows users to develop a personalized quit plan, identify smoking triggers, and upload a photo of their inspiration to quit. Step 2, “Be Aware,” contains 3 exercises to illustrate the problems with trying to control thoughts, feelings, and physical sensations rather than allowing them to come and go. Step 3, “Be Willing,” contains 8 exercises to help users practice allowing thoughts, feelings, and physical sensations that trigger smoking. Step 4, “Be Inspired,” contains 15 exercises to help participants identify deeply held values inspiring them to quit smoking and exercise self-compassion in response to smoking lapses.

#### Smokefree.gov

The US NCI Smokefree.gov website follows the USCPG and provides a standard treatment that teaches skills to smokers to avoid urges. Users can navigate through all pages of the website at any time, and there are no restrictions on the order in which the content can be viewed. Smokefree has 3 main sections: “Quit today,” “Preparing to quit,” and “Smoking issues.” The “Quit today” section has 7 pages of content that provide tips for the quit day, staying smoke free, and dealing with cravings. This section also provides information on withdrawal and the benefits of quitting. The “Prepare to quit” section has 7 content pages providing information on various reasons to quit, what makes quitting difficult, how to make a quit plan, and using social support during a quit attempt. The “Smoking issues” section provides 5 pages on the health effects of smoking and quitting, depression, stress, and secondhand smoke, and coping with the challenges of quitting smoking for the lesbian, gay, bisexual, and transgender community.

### Measures

#### Daily Log-in Count Predictors

Engagement with the assigned digital intervention was objectively measured using either Google Analytics (in the iCanQuit trial) or an internally hosted secure server (in the WebQuit trial). For each participant, time- and date-stamped log file records of opening each digital program were recorded. For the main analysis, we used the number of log-ins on each of the first 7 days following randomization as the predictor variables (ie, 7 different log-in variables, with 1 for each of the first 7 days after randomization).

#### Baseline Characteristic Predictors

The 13 self-reported baseline characteristics were (1) age, (2) gender, (3) race, (4) Hispanic ethnicity, (5) highest education level, (6) working status (full- or part-time employment vs any other), (7) sexual orientation, (8) whether they smoked half a pack of cigarettes per day (≤10 vs ≥11), (9) smoking within 5 minutes of waking (yes or no), (10) use of e-cigarettes in the past 30 days (yes or no), (11) confidence in being smoke free, (12) heavy drinking [35], and (13) current self-report of depression. For iCanQuit and QuitGuide, confidence in being smoke free was a single-item self-report measure on a scale from 0 to 100; for WebQuit and Smokefree, confidence was measured using the Commitment to Quitting Smoking Scale ranging from 1 to 5 [36]. Heavy drinking was defined as women who reported consuming ≥4 alcoholic drinks on a typical drinking day and men who reported consuming ≥5 alcoholic drinks on a typical drinking day [37]. These baseline variables were chosen as potential predictors of early dropout as they predicted engagement trajectory group membership in our previous studies and are commonly collected in digital intervention research [24,25].

#### Early Dropout Outcome

As reported in our previous research [24,25], early dropouts were defined as users who were categorized as “one-week users” in each intervention using functional clustering analysis of log-in trajectories [38,39]. The proportion of early dropouts was 57.06% (610/1069) for iCanQuit, 65.32% (695/1064) for QuitGuide, 55% (682/1240) for WebQuit, and 49.27% (645/1309) for Smokefree.

### Statistical Analyses

#### Missing Data

We assessed the level of missing data for both the baseline predictor variables and early dropout outcome. In our previous studies of app and website use trajectories, the proportion of participants missing use data ranged from 0.7% to 12%; these participants could not be categorized as 1-week users or longer-term users and were excluded from this study. Of the baseline variables used in this study, only heavy drinking status was missing for some participants in the iCanQuit and QuitGuide data (31/1069, 2.9% and 31/1064, 2.91% missing, respectively). The variables race, confidence in being smoke free, and heavy drinking status had missing values for some participants in the WebQuit and Smokefree data (48/1240, 3.87% and 43/1309, 3.28% missing overall, respectively). Given these small proportions, participants with missing values were excluded from the analysis. Thus, the final sample sizes for this study were 1038, 1033, 1192, and 1266 for the iCanQuit, QuitGuide, WebQuit, and Smokefree arms, respectively. Analyses were conducted using R (version 4.1.3; R Foundation for Statistical Computing) [40], and statistical tests were 2-sided with α=.05.

#### Comparison of Classification Models

For the first step in the analysis, we compared 4 of the most common statistical approaches to classification problems: standard multivariate logistic regression, decision tree, support vector machine (SVM), and neural network models [41-43]. Logistic regression has the advantage of being easily interpretable, but a downside is that the classifier derived from logistic regression can only achieve the Bayes rule if it is linear in the covariates included in the model. Decision trees are useful when the positives and negatives are only partially linearly separable. SVM is useful when one wishes to build a computationally efficient classifier using high-dimensional basis expansions of the covariates. Neural networks can capture the complex relationships between predictor variables and outcomes.

Each classification method was implemented to predict early dropout based only on log-in counts from days 1 to 7 after randomization. For each intervention, a training data set was used to fit the classification model, whereas the test data set was used to assess the performance of the model. Across all arms of the trials, 80% (3623/4529) of the participants were randomly selected into the training data set, and the remaining 20% (906/4529) were used as the test data set. The classification models were implemented using the Python package *sklearn* (Python Software Foundation) [44]. Tuning parameters were selected by 10-fold cross-validation, including the minimum number of samples (selected among 1, 10, 30, or 50) required to be at a leaf node in the decision tree, the regularization parameter (selected among 0.1, 1, 10, or 100) in SVM, and the hidden layer sizes (ie, 2 hidden layers, and the number of neurons in each layer was selected among 2, 3, or 4) in neural networks. All other parameters used were default values in *sklearn*. The basis expansion used for SVM was the radial basis. The models for each arm were fitted independently.

The receiver operating characteristic curve and corresponding area under the curve (AUC) were used to evaluate the performance of each predictive classification model [45]. AUC values range from 0.0 to 1.0, with a value of 1.0 indicating that the model perfectly classifies positive and negative outcomes (ie, early dropout vs longer-term user) across all classifier thresholds. In contrast, an AUC value of 0.0 indicates that the model predicts all negatives as positives and all positives as negatives. An AUC value of 0.5 means that the model does not perform better than random chance as it ranks a random positive case lower than a random negative case 50% of the time. In general, an AUC value between 0.7 and 0.8 is considered acceptable, 0.8 to 0.9 is considered excellent, and >0.9 is considered outstanding [46]. AUC values were compared between models using the “roc.test” function in the R library *pROC* [47].

#### Comparison of Logistic Regression Models

Next, we assessed whether the inclusion of baseline variables in the logistic regression could improve the prediction of early dropout. All 13 baseline variables (see the Baseline Characteristic Predictors section for a complete list) were added to the log-in counts from days 1 to 7 in the full model. Finally, we assessed whether a more parsimonious logistic regression model could improve the prediction of early dropout. In this model, the 13 baseline variables were added to the log-in counts of days 1 to 7 as inputs to stepwise logistic regression using the Bayesian information criterion for variable selection [48,49]. As with the comparison of classification methods, the 3 logistic regression models were fitted to the training data, and their performance was compared using AUC values from the test data to select a final model. The sensitivity, specificity, positive predictive value, and negative predictive value were calculated for the final model for each intervention.

## Results

### Baseline Characteristics of Each Digital Intervention Treatment Group

The baseline characteristics of each digital intervention treatment group are reported in Table 1. Participants in the website trial (WebQuit vs Smokefree) had very similar baseline characteristics, as did participants in the smartphone trial (iCanQuit vs QuitGuide). Compared with participants in the website trial (WebQuit vs Smokefree), participants in the smartphone trial (iCanQuit vs QuitGuide) were descriptively younger (mean age 37.91, SD 10.76 years vs 46.39, SD 13.30 years), and there was a higher percentage of male participants (304/1038, 29.29% and 304/1033, 29.33% vs 232/1192, 19.46% and 261/1266, 20.62%); a higher percentage of minority race (366/1038, 35.26% and 371/1033, 35.91% vs 294/1192, 24.66% and 348/1266, 27.49%); a higher percentage of participants with a high school or lower education (419/1038, 40.37% and 414/1033, 40.08% vs 332/1192, 27.85% and 348/1266, 27.49%); a higher percentage of lesbian, gay, or bisexual participants (188/1038, 18.11% and 174/1033, 16.84% vs 108/1192, 9.06% and 130/1266, 10.27%); a higher percentage of nicotine-dependent positive screen results (ie, first cigarette within 5 minutes of waking up; 556/1038, 53.56% and 560/1033, 54.21% vs 504/1192, 42.28% and 516/1266, 40.76%); a lower percentage of e-cigarette users (259/1038, 24.95% and 234/1033, 22.65% vs 415/1192, 34.82% and 441/1266, 34.83%); and a higher percentage of participants reporting heavy drinking (161/1038, 15.51% and 146/1033, 14.13% vs 130/1192, 10.91% and 140/1266, 11.06%). Descriptive statistics for the number of log-ins on each of the first 7 days for each intervention are shown in Table S1 in Multimedia Appendix 1.

**Table 1:** Baseline characteristics of participants assigned to each digital intervention (N=4529).

	iCanQuit (n=1038)	QuitGuide (n=1033)	WebQuit (n=1192)	Smokefree (n=1266)
Age (years), mean (SD)	37.92 (10.70)	37.89 (10.82)	46.54 (13.34)	46.25 (13.27)
Male gender, n (%)	304 (29.3)	303 (29.3)	232 (19.5)	261 (20.6)
Hispanic ethnicity, n (%)	97 (9.3)	93 (9)	90 (7.6)	118 (9.3)
Minority race, n (%)	366 (35.3)	371 (35.9)	294 (24.7)	348 (27.5)
High school or lower education, n (%)	419 (40.4)	414 (40.1)	332 (27.9)	348 (27.5)
Working, n (%)	575 (55.4)	575 (55.7)	621 (52.1)	654 (51.7)
Lesbian, gay, or bisexual, n (%)	188 (18.1)	174 (16.8)	108 (9.1)	130 (10.3)
Current depression, n (%)	299 (28.8)	305 (29.5)	300 (25.2)	362 (28.6)
Smokes ≥11 cigarettes per day, n (%)	764 (73.6)	780 (75.5)	942 (79)	999 (78.9)
First cigarette within 5 minutes of waking, n (%)	556 (53.6)	560 (54.2)	504 (42.3)	516 (40.8)
Used e-cigarettes at least once in the past month, n (%)	259 (25)	234 (22.7)	415 (34.8)	441 (34.8)
Confidence in being smoke-free^a^, mean (SD)	63.85 (27.04)	64.92 (26.62)	3.99 (0.74)	4.00 (0.77)
Heavy drinker^b^, n (%)	161 (15.5)	146 (14.1)	130 (10.9)	140 (11.1)

^a^Different scales were used. For iCanQuit and QuitGuide, the range was 0-100; for WebQuit and Smokefree, the range was 1-5.

^b^*Heavy drinker* was defined as women who reported consuming ≥4 drinks and men who reported consuming ≥5 drinks on a typical drinking day.

### Comparison of Classification Models Using AUC

For the iCanQuit test data, the AUC values were 0.94 (95% CI 0.90-0.97) for the logistic regression model, 0.92 (95% CI 0.88-0.96) for the decision tree, 0.93 (95% CI 0.89-0.97) for SVM, and 0.94 (95% CI 0.90-0.97) for the neural network model. The results of these analyses, along with those from the QuitGuide, WebQuit, and Smokefree data sets, are shown in Table 2. Compared with the standard logistic regression model, the decision tree, SVM, and neural network models did not significantly improve the AUC for iCanQuit or any of the other 3 digital interventions. For this reason, and as the standard logistic regression model is accessible and straightforward to implement, only logistic regression was used in all subsequent analyses.

**Table 2:** Performance, as measured by the area under the curve (AUC), and comparison of the 4 different models examined for each intervention.

Model and data set		iCanQuit, AUC (95% CI)	QuitGuide, AUC (95% CI)	WebQuit, AUC (95% CI)	Smokefree, AUC (95% CI)
**Logistic regression^a^**					
	Training	0.93 (0.91-0.95)	0.88 (0.86-0.91)	0.82 (0.80-0.85)	0.59 (0.56-0.62)
	Test	0.94 (0.90-0.97)	0.88 (0.83-0.93)	0.85 (0.80-0.90)	0.60 (0.54-0.66)
**Decision tree**					
	Training	0.95 (0.93-0.96)	0.87 (0.85-0.90)	0.83 (0.80-0.86)	0.60 (0.58-0.63)
	Test	0.92 (0.88-0.96)^b^	0.87 (0.82-0.92)^c^	0.84 (0.80-0.89)^d^	0.60 (0.54-0.66)^e^
**Support vector machine**					
	Training	0.93 (0.92-0.95)	0.88 (0.86-0.91)	0.83 (0.80-0.85)	0.61 (0.58-0.64)
	Test	0.93 (0.89-0.97)^f^	0.87 (0.82-0.92)^g^	0.83 (0.78-0.89)^d^	0.59 (0.54-0.65)^h^
**Neural network**					
	Training	0.93 (0.91-0.95)	0.87 (0.85-0.90)	0.83 (0.80-0.85)	0.57 (0.54-0.60)
	Test	0.94 (0.90-0.97)^i^	0.88 (0.83-0.93)^j^	0.85 (0.80-0.90)^d^	0.59 (0.53-0.65)^k^

^a^Each *P* value listed is for the comparison with the logistic regression model evaluated on the test data set.

^b^*P*=.11.

^c^*P*=.40.

^d^*P*=.29.

^e^*P*=.56.

^f^*P*=.23.

^g^*P*=.25.

^h^*P*=.91.

^i^*P*=.44.

^j^*P*=.78.

^k^*P*=.63.

### Comparison of Logistic Regression Models Predicting Early Dropout

As shown in Table S2 in Multimedia Appendix 1, the inclusion of the 13 baseline variables in the logistic regression models did not significantly improve the AUC for the iCanQuit data or any of the other 3 digital interventions. The logistic regression models resulting from stepwise selection using the Bayesian information criterion in each intervention included log-in counts only; no baseline variables remained in the selected models. For the iCanQuit training data, the variables selected by stepwise logistic regression were the log-in counts from the second to the seventh day after randomization. A similar result was obtained for the WebQuit data. Stepwise regression retained log-in counts for all 7 days after randomization for the QuitGuide data and for days 1 to 2 for the Smokefree data. The AUC for the iCanQuit stepwise regression model was 0.94 (95% CI 0.90-0.97) and was not significantly different from the AUC for the first logistic regression model using log-in counts from days 1 to 7 (*P*=.62). The results were similar for the other 3 digital therapeutic data sets. No baseline variables predicted early dropout across any of the 4 interventions, as shown in Table S3 in Multimedia Appendix 1. There were only a small number of baseline variables that predicted early dropout for 1 intervention but for no other. Thus, the final model selected to predict early dropout in each digital intervention was the logistic regression model using the first 7 days of log-in count data.

### Sensitivity and Specificity of the Final Models

At the standard classification threshold of 0.5, the sensitivity and specificity of the test data were 0.91 and 0.85, respectively, for iCanQuit; 0.90 and 0.64, respectively, for QuitGuide; 0.95 and 0.55, respectively, for WebQuit; and 0.55 and 0.58, respectively, for Smokefree. By decreasing the classification threshold to 0.3, the sensitivity increased to ≥0.95 for all interventions, whereas the specificity decreased. These results are presented in Table 3 along with the corresponding miss rates, positive predictive values, and negative predictive values.

**Table 3:** Model performance metrics for the final logistic regression models using classification thresholds of 0.3, 0.5, and 0.7.

		iCanQuit	QuitGuide	WebQuit	Smokefree
**Classification threshold=0.3**					
	Sensitivity	0.95	0.96	0.98	0.96
	Specificity	0.76	0.52	0.47	0.10
	Miss rate	0.05	0.04	0.02	0.04
	PPV^a^	0.83	0.79	0.67	0.54
	NPV^b^	0.92	0.88	0.96	0.71
	True positives, n	109	129	122	128
	False positives, n	22	35	61	109
	True negatives, n	71	38	54	12
	False negatives, n	6	5	2	5
**Classification threshold=0.5**					
	Sensitivity	0.91	0.90	0.95	0.55
	Specificity	0.85	0.64	0.55	0.58
	Miss rate	0.09	0.10	0.05	0.45
	PPV	0.88	0.82	0.69	0.59
	NPV	0.89	0.78	0.91	0.54
	True positives, n	105	121	118	73
	False positives, n	14	26	52	51
	True negatives, n	79	47	63	70
	False negatives, n	10	13	6	60
**Classification threshold=0.7**					
	Sensitivity	0.82	0.82	0.74	0.01
	Specificity	0.88	0.77	0.75	0.98
	Miss rate	0.18	0.18	0.26	0.99
	PPV	0.90	0.87	0.76	0.33
	NPV	0.80	0.70	0.73	0.47
	True positives, n	94	110	92	1
	False positives, n	11	17	29	2
	True negatives, n	82	56	86	119
	False negatives, n	21	24	32	132

^a^PPV: positive predictive value.

^b^NPV: negative predictive value.

## Discussion

### Aims and Principal Findings

The overall aim of this study was to identify a small number of highly generalizable and easily collected variables that can predict early dropout with high precision using statistical models that can be readily implemented by other researchers and intervention developers. To address this aim, this study explored whether log-in count data using standard statistical methods can precisely predict whether an individual will become an iCanQuit early dropout (ie, 1-week user) while validating the approach using the randomized trial data from 3 other digital interventions for smoking cessation, namely, QuitGuide, WebQuit.org, and Smokefree.gov. We also examined whether a limited number of commonly used self-reported baseline demographic variables (eg, age and education) might improve this prediction. Overall, the results showed that the AUC for each logistic regression model using only the first 7 days of log-in count variables was 0.94 (95% CI 0.90-0.97) for iCanQuit, 0.88 (95% CI 0.83-0.93) for QuitGuide, 0.85 (95% CI 0.80-0.88) for WebQuit.org, and 0.60 (95% CI 0.54-0.66) for Smokefree.gov. Replacing logistic regression models with the more complex methods of decision trees, SVM, or neural network models did not significantly increase the AUC, nor did including additional baseline variables as predictors. The sensitivity and specificity were generally good, and they were excellent for iCanQuit.

The results generally supported the overall study aim, showing that the first 7 days of log-in count data were sufficient to predict early dropout from a digital intervention. This prediction was achieved using a standard logistic regression model and validated with large randomized trial data sets testing 3 other digital interventions. Indeed, the pattern of results was generally robust across different clinical content (ie, ACT vs USCPG), structure of intervention delivery (ie, sequential vs parallel), and delivery platforms (ie, smartphone app vs website). The final models showed that a higher number of log-in counts in the first 7 days after randomization predicted a lower likelihood of being an early dropout, as demonstrated by the significant negative coefficients. The pattern of results was generally more pronounced for the log-ins occurring on later days of the week, which showed higher-magnitude negative coefficients. For example, in the iCanQuit data, the number of log-ins on days 5, 6, and 7 was especially predictive of being an early dropout. Across the 4 interventions, the number of log-ins on day 7 was generally the strongest significant predictor of early dropout.

### Comparison With Prior Work

This study provides 2 major advances on previous research on predicting early dropout in digital interventions. The first advance is that 1 type of variable that is straightforward, objective, and commonly measured provided reliable prediction of early dropout as compared with previous studies that have used a complex set of variables that are difficult to generalize and replicate (eg, specific intervention user journeys) or have relied on self-reporting, which requires extra resources to collect, has higher missing data rates, and has subjective measurement biases [12-20]. In this study, only 1 type of variable was sufficient for prediction, as opposed to requiring multiple variables as found in previous studies. The second key advance is that the statistical model that best predicted early dropout was a standard logistic regression model, as opposed to more sophisticated machine learning models that require more advanced knowledge of machine learning as well as large amounts of data, have greater computational burden, and are prone to overfitting [50]. Thus, the logistic regression approach of this study (vs more sophisticated statistical analysis methods) is more replicable and accessible to other researchers. As far as we are aware, this is one of the few studies to use log-in count data as a predictor of early dropout from digital health interventions [15,20]. A small body of research in the domain of gaming has examined the prediction of early dropout [51,52]. Although this domain is difficult to compare with digital health interventions, there are notable parallels. For example, in an analysis of casual gaming data, Kim et al [51] evaluated 5 different machine learning algorithms to predict game player churn (ie, dropout) in web-based and mobile games and found that, although prediction performance evaluated using AUC had little dependence on the choice of algorithm, shorter time playing and fewer number of play sessions predicted churn. Overall, this study lends support to a more straightforward approach of a single class of predictor (ie, log-in count data) and a statistical analysis method (ie, standard logistic regression) that may help the field make more rapid advances in the study of digital intervention dropout prediction.

### Future Directions

To build on this study’s findings, future research could focus on replicating this approach in other digital intervention data sets as well as testing adaptive treatment augmentations for early dropouts. Specifically, replications of the model could focus on an array of possible digital intervention platforms (eg, apps, chatbots, websites, and SMS text messaging), types of behavior change interventions (eg, diet, exercise, and medication management), and types of study design (eg, real-world use data, prospective research studies, and randomized trials). In an SMS text messaging context, an analogous engagement metric to “daily login” would be a time-stamped SMS text message engagement with the user (ie, the user sends an SMS text message or otherwise engages with the SMS text messaging platform). Regarding adaptive interventions, this study has implications for developing a randomized trial that offers an adapted, augmented intervention for individuals who are likely to drop out of their assigned digital intervention. The Sequential Multiple Assignment Randomized Trial is an increasingly common type of factorial design that could be applied to the problem of early dropout from a digital intervention by experimentally manipulating the offer (vs not) of a specific treatment augmentation [53-55]. The results of these studies might indicate what form this augmentation might take, such as sending special push notification messages, providing incentives to log-in, or offering outreach phone calls. The results of this study provide a guide for developing a decision rule for when to offer an augmented intervention to digital intervention study participants at risk of dropping out. The study results on the classification thresholds can assist researchers in deciding whether to set lower versus higher thresholds for the decision rule. For example, a threshold of 0.3 would capture more early dropouts but comes at the increased cost of offering treatment augmentation for more people, whereas a threshold of 0.7 would capture fewer dropouts and, thus, require less cost to provide augmentation but comes at the risk of missing more people who are likely to drop out. Given that a fraction of 1-week users will quit smoking without an augmented intervention, there is a risk that extra resources will be devoted to these individuals who, ultimately, would not need them. In general, that is always a consequence of early intervention for at-risk individuals—some will go on to be ultimately successful, and it can be difficult to predict who those individuals will be. Thus, an additional intervention may be a modest cost of resources when the net benefit will be a boost in the overall success of the entire group of early dropouts. Depending on the costs and benefits of the augmented intervention, one classification threshold might make more sense than another.

### Limitations

This study has important limitations. First, as fitting the study aims, the study was an exploratory analysis as participants were not randomized to log-in a different number of times each day. Thus, caution should be taken regarding making causal inferences from the analysis. Second, because of a Google Analytics error, there was approximately 11% of missing use data for the iCanQuit trial. Finally, the model performance predicting early dropout was generally weakest for the NCI Smokefree digital intervention. Although there is nothing unusual about this intervention in terms of its content or structure, it would be worthwhile for future research to conduct diagnostics into the features of this intervention to learn why model performance was weaker for the prediction of early dropouts. For example, our previous research identified 3 log-in trajectory groups in Smokefree: 1-week (ie, early dropout), 4-week, and 5-week users [24]. However, further inspection showed a small difference in terms of log-ins per week between the 1-week and 4-week user groups. This small difference likely made it difficult for the statistical models to predict Smokefree early dropouts, thereby lowering the AUC values for Smokefree. We suspect that this may have led to the models predicting early dropout (ie, 1-week users) for the Smokefree intervention having a weaker performance than the models for the other 3 digital interventions.

### Strengths

This study has important strengths. First, the sample size for each digital intervention was large (range 1038-1266), with a total combined sample size of 4529. Second, the sample was geographically diverse, recruited from all 50 US states, and had a high percentage of participants with minority race and ethnic backgrounds. Third, the type of variable that predicted early dropout was objectively collected, thereby reducing measurement bias. Related, the predictor variable is commonly collected by other researchers, thus making the analyses more comparable and readily replicable. Fourth, the pattern of results found in the primary sample of the iCanQuit data was validated in 3 other digital interventions with different clinical content (ie, ACT vs USCPG), structure of intervention delivery (ie, sequential vs parallel), and delivery platforms (ie, smartphone app vs website). Finally, a highly generalizable quality of these trials is that they were conducted in the real world (as opposed to a laboratory), where users were completely free to log-in at will and in the context of their daily lives.

### Conclusions

In conclusion, logistic regression models using only the first 7 days of log-in count data are generally good at predicting early dropouts. These models performed well when using simple, automated, and readily available log-in count data, whereas including self-reported sociodemographic baseline variables did not improve the prediction. The results will inform the early identification of people at risk of early dropout from digital health interventions with the goal of intervening further by providing them with augmented treatments to increase their retention and, ultimately, their intervention outcomes.

## Appendix

Multimedia Appendix 1 Supplementary tables.

Multimedia Appendix 2 Analysis data sets.

## Abbreviations

ACT: acceptance and commitment therapy
AUC: area under the curve
NCI: National Cancer Institute
RCT: randomized controlled trial
SVM: support vector machine
USCPG: United States Clinical Practice Guidelines

## Data Availability

All the data that were analyzed in this study are available in Multimedia Appendix 2.
