# Supplementary material for: Can a Single Variable Predict Early Dropout From Digital Health Interventions? Comparison of Predictive Models From Two Large Randomized Trials
Source: J Med Internet Res. 2023 Jan 20;25:e43629. doi: 10.2196/43629 (PMC9898835; doi:10.2196/43629)
Supplement: Multimedia Appendix 1 [file jmir_v25i1e43629_app1.docx]

This is a Multimedia Appendix to a full manuscript published in the J Med Internet Res. For full copyright and citation information see <http://dx.doi.org/10.2196/jmir.43629>

**Supplementary Table 1:** Mean (SD) and median (interquartile range) number of logins on each of the first seven days for each intervention.

|  | iCanQuit | QuitGuide | WebQuit | Smokefree |
| --- | --- | --- | --- | --- |
| Day 1 | 2.34 (1.58) 2 (1-3) | 1.66 (0.95) 1 (1-2) | 1.54 (0.95) 1 (1-2) | 1.31 (0.64) 1 (1-1) |
| Day 2 | 1.05 (1.41) 1 (0-2) | 0.32 (0.66) 0 (0-0) | 0.35 (0.73) 0 (0-1) | 0.10 (0.33) 0 (0-0) |
| Day 3 | 0.80 (1.26) 0 (0-1) | 0.20 (0.50) 0 (0-0) | 0.24 (0.65) 0 (0-0) | 0.08 (0.34) 0 (0-0) |
| Day 4 | 0.68 (1.17) 0 (0-1) | 0.15 (0.50) 0 (0-0) | 0.20 (0.65) 0 (0-0) | 0.05 (0.31) 0 (0-0) |
| Day 5 | 0.61 (1.13) 0 (0-1) | 0.12 (0.38) 0 (0-0) | 0.19 (0.68) 0 (0-0) | 0.04 (0.26) 0 (0-0) |
| Day 6 | 0.59 (1.23) 0 (0-1) | 0.11 (0.40) 0 (0-0) | 0.18 (0.64) 0 (0-0) | 0.04 (0.29) 0 (0-0) |
| Day 7 | 0.59 (1.22) 0 (0-1) | 0.14 (0.44) 0 (0-0) | 0.14 (0.47) 0 (0-0) | 0.04 (0.26) 0 (0-0) |

**Supplementary Table 2:** Performance, as measured by AUC, of logistic regression models predicting early dropout from daily login counts and baseline variables.

| Model and data set | iCanQuit | | QuitGuide | | WebQuit | | Smokefree | |
| --- | --- | --- | --- | --- | --- | --- | --- | --- |
|  | AUC  (95% CI) | *P* value | AUC  (95% CI) | *P* value | AUC  (95% CI) | *P* value | AUC  (95% CI) | *P* value |
| Daily login count only | 0.94  (0.90-0.97) | Ref. | 0.88  (0.83-0.93) | Ref. | 0.85  (0.80-0.88) | Ref. | 0.60  (0.54-0.66) | Ref. |
| Daily login count plus baseline variables | 0.93  (0.89-0.97) | .18 | 0.86  (0.81-0.92) | .13 | 0.84  (0.79-0.89) | .46 | 0.58  (0.51-0.65) | .59 |
| Variables selected by stepwise regression^a^ | 0.94  (0.90-0.97) | .62 | 0.88  (0.83-0.93) | >.99 | 0.83  (0.78-0.88) | .16 | 0.58  (0.53-0.64) | .35 |

^a^The variables selected as the best predictors of early dropout by stepwise regression using BIC were: (a) number of logins on days 2-7 (iCanQuit); (b) number of logins on days 1-7 (QuitGuide); (c) number of logins on days 2-7 (WebQuit); and (d) number of logins on days 1-2 (Smokefree).

**Supplementary Table 3.** Model coefficients for logistic regression models predicting early dropout, with both baseline variables and daily login count.

|  | iCanQuit | | QuitGuide | | WebQuit | | Smokefree | |
| --- | --- | --- | --- | --- | --- | --- | --- | --- |
|  | Estimate (SE) | *P* value | Estimate (SE) | *P* value | Estimate (SE) | *P* value | Estimate (SE) | *P* value |
| Age | -.001 (.01) | .96 | .01 (.009) | .19 | -.02 (.006) | .01 | -.007 (.005) | .12 |
| Male gender | -.31 (.23) | .17 | .20 (.21) | .33 | .23 (.19) | .21 | -.01 (.15) | .94 |
| Hispanic ethnicity | .29 (.38) | .44 | .33 (.33) | .32 | -.31 (.30) | .31 | -.32 (.24) | .17 |
| Minority race | .28 (.24) | .25 | -.12 (.21) | .58 | -.24 (.2) | .23 | -.11 (.16) | .49 |
| High school or less education | .24 (.21) | .26 | -.11 (.19) | .55 | .24 (.17) | .14 | .13 (.13) | .32 |
| Working | .13 (.20) | .51 | .30 (.19) | .11 | -.19 (.15) | .21 | .34 (.12) | .006 |
| Lesbian, gay, or bisexual | .10 (.27) | .72 | -.24 (.25) | .34 | -.13 (.25) | .59 | -.23 (.20) | .24 |
| Current depression | -.02 (.23) | .92 | .38 (.21) | .07 | .15 (.17) | .37 | .02 (.13) | .87 |
| Smokes >=11 cigarettes per day | .51 (.24) | .03 | -.005 (.21) | .98 | -.06 (.18) | .75 | .32 (.15) | .04 |
| First cigarette within  5 minutes of waking | .20 (.21) | .34 | -.06 (.18) | .75 | .34 (.15) | .03 | .04 (.12) | .73 |
| Used e-cigarettes at least once in past month | -.02 (.23) | .92 | -.05 (.22) | .81 | .29 (.15) | .06 | -.09 (.12) | .46 |
| Confidence in being smokefree | -.003 (.004) | .49 | .004 (.003) | .19 | .10 (0.10) | .31 | .03 (.08) | .65 |
| Heavy drinker | .10 (.29) | .74 | .07 (.26) | .80 | .17 (.23) | .47 | .42 (.19) | .03 |
| Day 1 | -.12 (.07) | .12 | -.60 (.10) | <.001 | -.14 (.09) | .13 | -.25 (.10) | .01 |
| Day 2 | -.41 (.10) | <.001 | -1.31 (.16) | <.001 | -.97 (.15) | <.001 | -.66 (.21) | .001 |
| Day 3 | -.39 (.13) | .002 | -1.90 (.22) | <.001 | -.64 (.19) | .001 | -.40 (.21) | .06 |
| Day 4 | -.79 (.15) | <.001 | -1.51 (.27) | <.001 | -.92 (.22) | <.001 | -.37 (.27) | .17 |
| Day 5 | -1.14 (.18) | <.001 | -2.09 (.29) | <.001 | -.78 (.22) | <.001 | .31 (.28) | .27 |
| Day 6 | -1.33 (.19) | <.001 | -1.67 (.29) | <.001 | -2.09 (.33) | <.001 | .31 (.27) | .25 |
| Day 7 | -1.22 (.19) | <.001 | -1.95 (.28) | <.001 | -1.99 (.38) | <.001 | -.55 (.29) | .06 |
